# Large‐Area Noise‐Resilient Multiplexing Triboelectric Biomechanical Sensing on Clothing for High‐Precision Full‐Body Motion Capture in Wearables

**DOI:** 10.1002/advs.77038

**Published:** 2026-08-06

**Authors:** Beibei Shao, Ming‐Han Lu, Wei‐Chen Peng, Wei‐Chun Yang, Yi‐Ting Chen, Jiun‐Wei Fong, Tzu‐Ching Lu, Tai‐Chen Wu, Zhi‐Xian Yan, Kai‐Yuan Hsiao, Jiann‐Yeu Chen, Ming‐Yen Lu, Heng‐Jui Liu, Ruiyuan Liu, Ying‐Chih Lai

**Affiliations:** ^1^ State Key Laboratory of Bioinspired Interfacial Materials Science, Institute of Functional Nano & Soft Materials (FUNSOM) Soochow University Suzhou Jiangsu People's Republic of China; ^2^ Department of Materials Science and Engineering National Chung Hsing University Taichung City Taiwan; ^3^ Department of Materials Science and Engineering National Tsing Hua University Hsinchu Taiwan; ^4^ Innovation and Development Center of Sustainable Agriculture, i‐Center for Advanced Science and Technology National Chung Hsing University Taichung City Taiwan; ^5^ College of Energy, Jiangsu Key Laboratory of Advanced Negative Carbon Technologies Soochow University Suzhou People's Republic of China

**Keywords:** full‐body motion capture, graphite‐like carbonized textiles, shielding layer, SnS_2_ nanoflowers, triboelectric sensing garment

## Abstract

Full‐body motion capture is vital for next‐generation wearables in virtual reality, the metaverse, and healthcare. However, current systems struggle to scale beyond localized sensing due to severe signal interference, power issues, and lack of scalable and stretchable sensing technology. Here, we develop a large‐area noise‐resilient multiplexed triboelectric sensing garment enabling active and high‐precision full‐body motion tracking. A multilayer architecture that applies shielding on both the sensing units and serpentine circuits largely suppresses signal misrecognition to 0.13%, attenuates electromagnetic noise by 57 dB, and raises threshold force to >1.5 N. Complementarily, the hybrid design of SnS_2_ nanoflowers (NFs)‐doped silicone rubber composites and SnS_2_ NFs‐decorated graphite‐like carbonized textiles markedly boost charge generation, transport, and retention, achieving high‐fidelity, noise‐free motion sensing. Further integrated with on‐body wireless processing and deep‐learning analytics, the system enables reliable acquisition and accurate recognition of multi‐site body motion simultaneously. It underpins immersive wearables, demonstrated in virtual gaming scenarios by precise upper‐body gesture recognition, lower‐limb action classification, and full‐body interactive control.

## Graphical abstract

A large‐area, noise‐resilient multiplexed triboelectric sensing garment based on a multilayer shielded architecture and SnS_2_ nanoflower‐engineered hybrid materials enables interference‐suppressed, high‐fidelity full‐body motion tracking, achieving 0.13% signal misrecognition, 57 dB electromagnetic noise attenuation, and >1.5 N threshold force, while supporting deep‐learning‐assisted multi‐site motion recognition for immersive wearables.

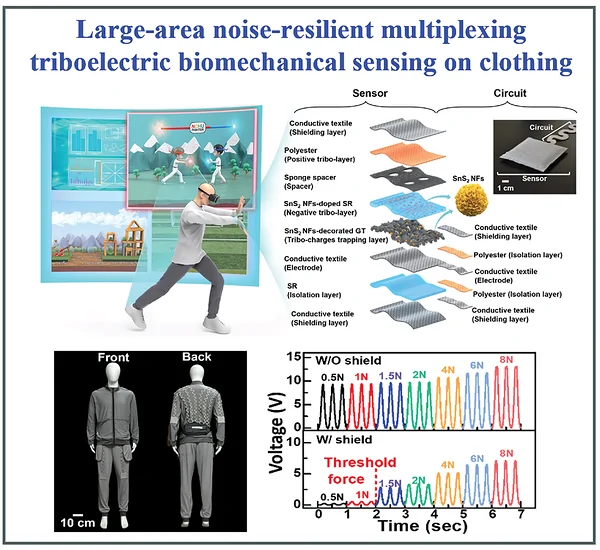

## 1 Introduction

Wearable electronics are reshaping human–machine interaction across virtual reality, immersive computing, and physiological monitoring [1, 2, 3, 4, 5]. As these applications transition toward the metaverse, an urgent demand emerges: accurate and interference‐free full‐body motion capture. Existing wearable systems, such as gloves or wristbands, can reliably track local motions, yet scaling these devices to whole‐body sensing remains a longstanding challenge [6, 7, 8, 9, 10, 11, 12, 13]. Optical full‐body motion‐tracking systems offer an alternative but suffer from intrinsic constraints, including frequent self‐occlusion, where body parts obstruct one another, and inherent privacy risks associated with continuous video capture in personal or public settings [14, 15, 16]. These constraints fundamentally restrict the deployment of optical methods in everyday metaverse or wearable scenarios.

A critical barrier to advancing wearable systems from localized sensing to full‐body motion capture is maintaining signal integrity across large‐area flexible circuits [17, 18, 19]. As the sensing area increases, wiring‐induced interference and ambient electromagnetic (EM) noise become pronounced [20, 21, 22], and full‐body configurations further amplify these issues through limb‐to‐limb coupling and signal blockage [22, 23, 24, 25]. Realizing whole‐body coverage requires multilayer, multiplexed circuit architectures with long routing distances and dense interconnect networks, which introduce parasitic capacitance, signal attenuation, and noise amplification, making reliable motion extraction increasingly difficult [26, 27, 28, 29]. While passive sensors can reduce wiring complexity, they depend on external power management that increases system weight, cost, and design burden [30, 31, 32]. Triboelectric nanogenerators (TENGs) stand out as an active sensing technology for self‐powered operation and strong biomechanical‐to‐electrical coupling [9, 10, 22]. However, when TENG sensors are scaled to garment‐ or body‐level arrays, severe misrecognition arises from interference among densely routed interconnects and surrounding EM noise [21, 22, 33, 34, 35, 36]. The resulting parasitic signals are easily mistaken for real outputs, undermining sensing fidelity and reliable full‐body motion capture.

Here, we present a multiplexing triboelectric sensing garment that supports self‐powered, high‐fidelity, wireless, and spatially synchronized motion capture across the entire body. It incorporates eight sensing nodes at key joint regions, together with stretchable serpentine circuits that conform to body mechanics and maintain reliable connectivity under motion. The deployments of shielding layers at both the sensing units and circuits yield electrostatically confined structures that markedly suppress wiring‐induced misrecognition to 0.13%, attenuate EM perturbations by 57 dB, and elevate threshold force to >1.5 N, ensuring faithful discrimination and positioning of genuine mechanical contact. Furthermore, the sensing node features a compact multilayer architecture incorporating SnS_2_ nanoflowers (NFs)‐doped silicon rubber (SR) composite and SnS_2_ NFs‐decorated graphite‐like carbonized textiles (GTs). This hybrid design maximizes charge generation, trapping, transport, and storage, enabling robust motion sensing. Coupled with an on‐body wireless processor and a deep‐learning (DL) backend, the sensing garment enables real‐time, temporally synchronized acquisition and recognition of full‐body signals from multiple sites. It also acquires accurate decoding of complex motions, reaching 94.6% for upper‐body gestures, 88.3% for lower‐limb actions, and 90.0% for full‐body gaming control. These advances establish a scalable, low‐cost, and interference‐resilient sensing framework for next‐generation wearables, enabling immersive metaverse interaction, rehabilitative monitoring, and embodied human–machine interfaces.

## 2 Results and Discussion

### 2.1 Wireless Full‐Body Multiplexing Triboelectric Sensing Garment

**FIGURE 1:**
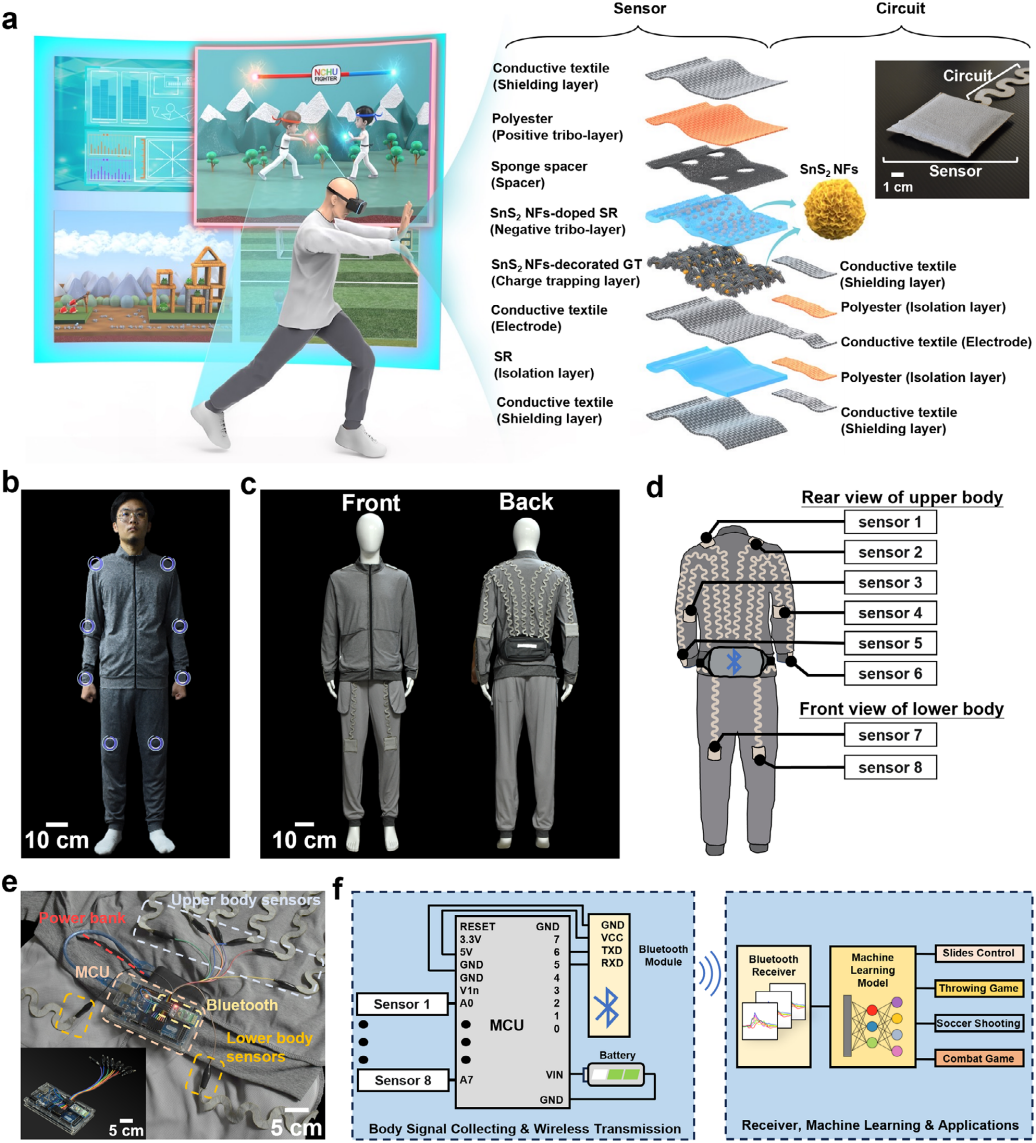
Application scenario, device architecture, and system configuration of large‐area multiplexed biomechanical‐sensing garment. (a) Schematic illustration of the sensing garment for metaverse and gaming applications. (b) Optical photograph of the sensing garment worn on human body. (c) Front and back views of the garment showing integrated sensing and wiring layout. (d) Schematic of multiplexed circuit configuration and sensor distribution. (e) Optical photograph of microcontroller unit and on‐garment signal‐processing module. (f) Schematic diagram of wireless data‐transfer pipeline and deep‐learning workflow for multichannel motion recognition.

Figure 1a illustrates the conceptual design of a metaverse‐oriented smart clothing comprising distributed and multiplexing sensing nodes integrating multilayered triboelectric sensors with stretchable circuits. A representative sensing unit occupies 7 × 7 cm^2^ with an overall thickness of 3 mm, enabling unobtrusive, garment‐scale deployment. The sensor module employs a hierarchical textile multilayer stack comprising an outer conductive‐fabric shielding layer, a polyester positive tribo‐layer, a sponge spacer for independent contact–separation, and an SnS_2_ NFs‐doped SR negative tribo‐layer (Figure S1). A SnS_2_ NFs‐decorated GT charge‐trapping layer, a conductive‐fabric electrode, an SR isolation layer, and a secondary conductive‐fabric shielding layer complete the assembly. The dual shielding layers play crucial roles in suppressing non‐contact electrostatic interference, eliminating false body‐contact recognition, and providing effective EM shielding, ensuring that only genuine triboelectric signals are captured during full‐body motion.

The circuit module incorporates serpentine interconnects encapsulated within a stratified textile stack comprising conductive‐fabric electrodes, polyester isolation layers, and dual conductive‐fabric shielding layers. These shielding layers prevent misidentified signals generated when human skin or objects contact the wiring, and provide EM immunity to ensure clean signal transmission in the multiplexed sensing array. Such shielding‐enabled circuit stability allows the sensing array to operate robustly under complex mechanical deformations, providing an essential foundation for scalable, artifact‐free, and multiplexed full‐body tactile interfaces.

The number and placement of the sensing nodes are fully reconfigurable and can be tailored to different interaction or monitoring scenarios. In the present demonstration, eight sensor modules are symmetrically distributed across the shoulders, elbows, wrists, and knees (Figure 1b,c), forming an ergonomically optimized layout that captures major joint motions while minimizing mechanical interference during daily activities. The front‐back layout of the textile interconnects enables efficient long‐distance signal routing while maintaining garment breathability and comfort (Figure 1d). The modular wiring architecture also allows additional sensors to be integrated along the serpentine pathways, scaling the system from localized joint monitoring to high‐density, garment‐level tactile or motion‐capture networks without redesigning the circuitry. The eight‐node layout was selected as a representative configuration that balances motion‐capture capability, wearing comfort, and wiring complexity. The sensing nodes were positioned at the shoulders, elbows, wrists, and knees because these major joints dominate the upper‐body gestures, throwing motions, lower‐limb kicking actions, and full‐body movements demonstrated in this work. The shoulder–elbow–wrist arrangement captures coordinated arm dynamics, while the knee sensors provide key lower‐limb motion information. Importantly, this layout is not fixed; the modular textile circuit design allows the number and placement of sensing nodes to be adjusted for different application scenarios or higher‐density full‐body motion tracking.

All sensing nodes are routed to a wearable processing unit positioned at the waist, which coordinates data acquisition and wireless transmission (Figure 1e). When body motion activates a triboelectric sensing event, the resulting signals travel through the stretchable interconnects and are digitized by the microcontroller unit, followed by wireless transmission through a Bluetooth module to an external receiver. The received multichannel data are then processed by a DL model that performs real‐time motion recognition and drives various metaverse and gaming applications, including slide control, throwing tasks, soccer shooting, and combat interaction (Figure 1f and Figure S2). To support continuous wireless operation, the system uses a 10 000 mAh power bank as the power source, and due to the active‐sensing characteristics of the triboelectric network, the garment can operate stably for more than one week.

Compared with previous triboelectric textile sensors, smart gloves, and tactile textiles that primarily focus on localized gesture or touch recognition, the present system addresses the signal‐interference challenge that emerges when triboelectric sensing networks are scaled to garment‐level full‐body motion capture. The key advance of this work is the system‐level shielding strategy applied simultaneously to both sensing nodes and long‐distance serpentine textile circuits. This dual‐level shielding architecture suppresses non‐contact electrostatic disturbance, circuit‐induced parasitic signals, and electromagnetic noise, thereby enabling large‐area multiplexed sensing with reduced signal misrecognition. Combined with wireless acquisition and DL‐assisted signal interpretation, this design establishes a noise‐resilient, garment‐scale triboelectric platform for reliable full‐body motion capture and interactive wearable applications. To further clarify the novelty and positioning of our wearable motion‐sensing system, we benchmarked it against representative state‐of‐the‐art TENG‐based systems in terms of sensing nodes, flexibility/stretchability, wireless capability, interference resilience, recognition accuracy, and full‐body control capability, as summarized in Table S1.

### 2.2 Structural Engineering and Performance of the Triboelectric Sensor

To enable an active triboelectric sensing mechanism with enhanced charge trapping, the negative tribo‐layer was engineered using petal‐like SnS_2_ NFs embedded in a rough‐surfaced SR matrix. The SnS_2_ NFs endowed with large specific surface areas act as efficient electrostatic charge collectors and transport pathways, rapidly directing charges toward the SnS_2_ NFs‐decorated GT [37, 38, 39]. The nanoflower morphology of SnS_2_ was deliberately selected rather than randomly chosen, as its hierarchical structure is beneficial for enhancing interfacial charge accumulation and retention. Compared with non‐hierarchical particles, the petal‐like architecture provides a larger effective contact area and more accessible edge/defect sites, which are favorable for triboelectric charge trapping. Therefore, the SnS_2_ nanostructure plays an active functional role in improving device performance instead of serving as a generic filler.

**FIGURE 2:**
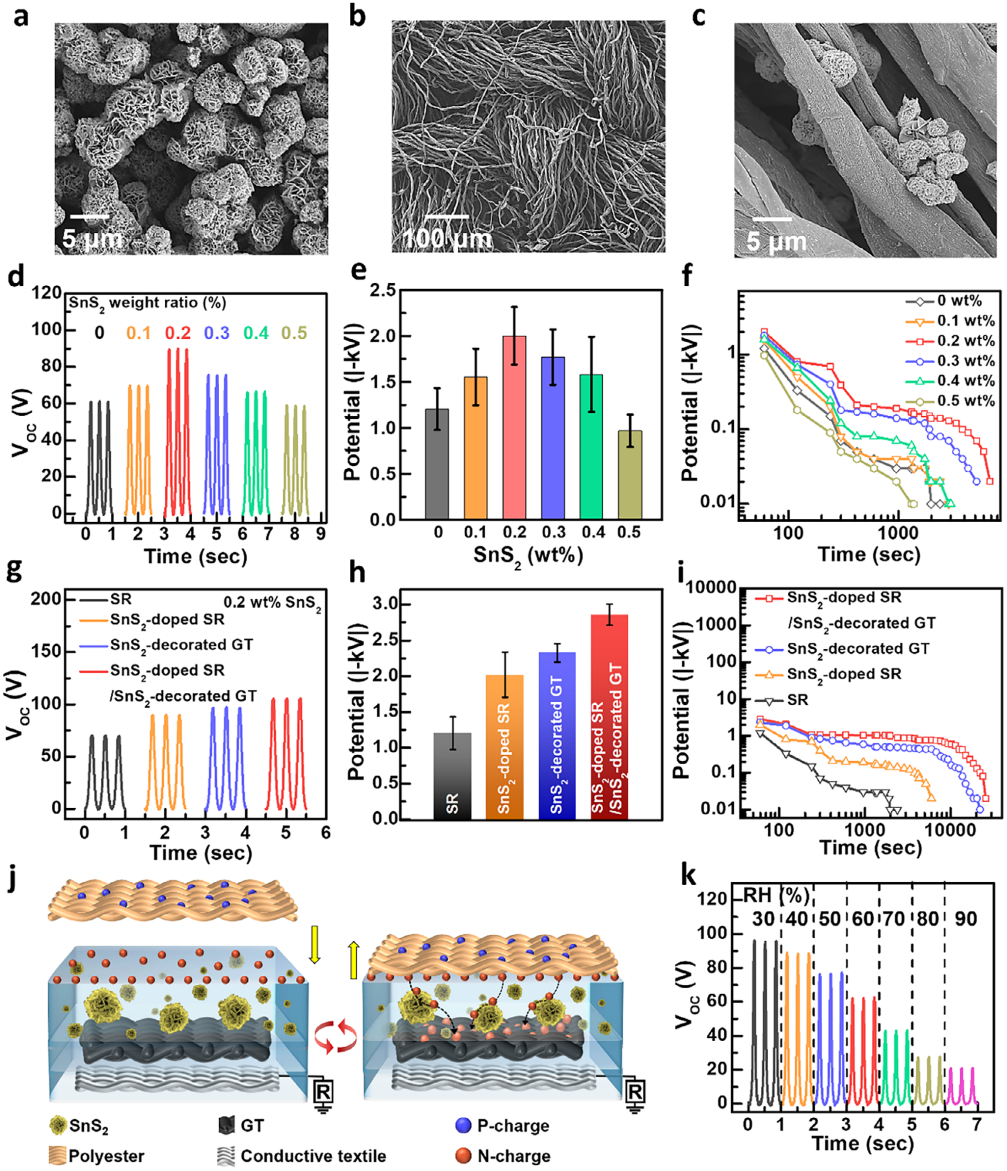
Fabrication, optimization, and performance of triboelectric sensor. SEM images of (a) synthesized SnS_2_ NFs, (b) GT, and (c) SnS_2_ NFs loaded on carbonized fiber. (d) Voltage, (e) surface potential–time, and (f) surface potential of device and materials with different weight ratios of SnS_2_ NFs. (g) Voltage, (h) surface potential–time, and (i) surface potential of devices with different structural configurations. (j) Schematic illustration of enhanced charge‐trapping mechanism of SnS_2_ NFs composite. (k) Voltage curves of device under different humidity.

In parallel, the SnS_2_ NFs‐decorated GT operates as a high‐capacity charge reservoir, where its extensive surface and abundant trapping states promote charge accumulation and suppress dissipation along the textile [39]. Scanning electron microscope (SEM) and energy dispersive X‐ray spectroscopy (EDS) analyses confirm that the SnS_2_ NFs exhibit a uniform sphere‐like morphology with homogeneous elemental distribution (Figure 2a and Figure S3). The X‐ray diffraction (XRD) patterns verify the crystalline SnS_2_ phase, with diffraction peaks matching the database results for the Berndite‐2T layered structure of SnS_2_ (JCPDS card No. 23‐0677) [11]. Transmission electron microscopy (TEM) reveals the hierarchical petal‐like structure with an overall diameter of 1.5–2 µm, while high‐resolution TEM (HRTEM) shows well‐defined lattice fringes with interplanar spacings of 0.19 and 0.28 nm, indicating high crystallinity favorable for charge retention (Figure S4). X‐ray photoelectron spectroscopy (XPS) spectra confirm the presence of Sn 3d and S 2p signals, with high‐resolution peaks at 163.0 and 161.8 eV (S 2p_1/2_ and S 2p_3/2_) and 494.7 eV and 486.3 eV (Sn 3d_3/2_, Sn 3d_5/2_), respectively (Figure S5).

The SnS_2_ NFs‐doped SR composite layer is patterned on rough abrasive paper to enlarge the effective contact area during cyclic contact‐separation, thereby increasing charge generation [10] (Figure S6). Incorporating SnS_2_ NFs into the dielectric SR matrix enables partial penetration of surface charges into the bulk of the triboelectric layer (Figure S7), which enhances the overall charge‐storage capacity and strengthens the local electrostatic field [12]. Importantly, this charge‐redistribution mechanism mitigates the sensitivity of the surface potential to ambient humidity, allowing the device to maintain stable and distinguishable electrical outputs even under perspiration‐induced moisture conditions [13, 37].

To further promote charge transfer toward the charge‐trapping layer, SnS_2_ NFs are deposited onto the GT, forming a hierarchical SnS_2_ NFs‐decorated interface that enhances charge‐storage efficiency (Figure 2b,c). SEM and EDS characterizations of the textile before and after carbonization reveal a pronounced transformation in textile morphology and composition (Figure S8). The pristine textile displays a smooth polymeric appearance, whereas high‐temperature carbonization produces a brittle, dehydrated structure with a markedly increased C:O ratio, shifting from 43.7:56.3 to 89.8:10.2, consistent with oxygen removal under an inert atmosphere. Raman spectra exhibit characteristic D and G bands at 1318.3 cm^−1^ and 1558.6 cm^−1^ (Figure S9), confirming the formation of graphitic domains [14]. XPS analysis further verifies progressive carbonization, showing C 1s and O 1s peaks at 285 eV and 532 eV, with deconvolution revealing C═C, C─C, C─O, and C═O bonding states indicative of partial graphitization (Figure S10). HR‐TEM imaging identifies an interlayer spacing of 0.35 nm, corresponding to the (002) plane of a sp^2^‐hybridized graphite‐like lattice (Figure S11), underscoring the atomic rearrangement responsible for improved electronic properties. As the foundation for both the shielding layers and the electrode network, the conductive textile must provide reliable long‐range charge transport. SEM and EDS analyses reveal that conductivity originates from densely interwoven silver fibers embedded within the textile matrix (Figure S12), offering a continuous percolation pathway essential for stable signal routing throughout the garment.

To elucidate the role of SnS_2_ NFs in enhancing active triboelectric sensing, devices incorporating different SnS_2_ NFs concentrations (0–0.5 wt%) were fabricated and evaluated. Both the open‐circuit voltage (*V*_OC_) and the short‐circuit current (*I*_SC_) increase markedly upon SnS_2_ NFs incorporation, with the highest output observed at 0.2 wt% (*V*_OC_ = 89.4 V, *I*_SC_ = 0.6 µA) (Figure 2d and Figure S13). Beyond this concentration, the performance declines, consistent with a percolation‐induced reduction in effective charge trapping. At 0.2 wt% SnS_2_ NFs, the composite exhibits the strongest charge‐retention behavior, achieving a maximum surface potential of ‐2 kV that persists for ≈115 min (Figure 2e,f). This enhancement originates from the increased charge density enabled by the elevated dielectric constant of the composite. According to the triboelectric theory, a higher dielectric constant promotes greater charge accumulation when the contact area and thickness are fixed [15]. The peak dielectric constant also occurs at 0.2 wt% SnS_2_ NFs (Figure S14), validating the strong structure‐property correlation. The optimized 0.2 wt% SnS_2_ NFs‐doped SR composite further demonstrates superior output performance when paired with polyester fabric, attributed to its favorable position in the triboelectric series and efficient charge transfer characteristics.

To evaluate the contribution of each structural component to active charge trapping, four configurations—pure SR, SnS_2_ NFs‐doped SR, SnS_2_ NFs‐doped SR/GT, and SnS_2_ NFs‐doped SR on SnS_2_ NFs‐decorated GT (SnS_2_ NFs‐doped SR/SnS_2_ NFs‐decorated GT)—were fabricated using 0.2 wt% SnS_2_ NFs and polyester fabric as the positive tribo‐material. Among them, the SnS_2_ NFs‐doped SR/SnS_2_ NFs‐decorated GT device delivers the highest output, achieving a *V*_OC_ of 104.2 V and an *I*_SC_ of 0.8 µA (Figure 2g and Figure S15). This enhancement correlates with the elevated surface potential and prolonged charge retention observed in the composite structures (Figure 2h,i). Incorporating the GT introduces an effective charge‐storage layer that significantly extends charge decay time. The SnS_2_ NFs‐doped SR/GT device maintains surface potential for ≈370 min, whereas the SnS_2_ NFs‐doped SR/SnS_2_ NFs‐decorated GT further prolongs retention to ≈460 min. These results indicate that both SnS_2_ NFs doping and the introduction of GT synergistically strengthen the material's charge‐trapping and retention capability. During each contact‐separation cycle, triboelectric charges are efficiently transferred through the SnS_2_ NFs‐based conductive pathways into the GT, where they are stored within the graphite‐like domains (Figure 2j and Figure S16). This hierarchical charge‐transfer architecture increases the effective charge‐accumulation capacity, reinforces the local electrostatic field, and consequently yields superior electrical output. However, it should be noted that the proposed charge‐transfer process is primarily supported by surface‐potential measurements (Figure 2e,f,h,i), which reflect enhanced charge accumulation and prolonged charge retention rather than direct microscopic charge‐transfer pathways. Therefore, the energy band diagram (Figure S17) is intended only as a qualitative illustration of a possible charge‐transfer tendency based on relative energy levels. The experimentally supported conclusion is the improved charge‐trapping and charge‐retention capability enabled by the SnS_2_ NFs/GT hierarchical structure.

Even under high humidity levels of up to 90% *RH*, the device preserves a *V*_OC_ of ≈20 V and an *I*_SC_ of ≈0.2 µA (Figure 2k and Figure S18). Such humidity tolerance is essential for wearable systems, which must operate reliably during heavy perspiration or in highly humid environments. The stable output demonstrates that the synergistic SnS_2_ NFs‐enhanced charge‐trapping and SnS_2_ NFs‐decorated GT charge‐storage architecture effectively mitigates moisture‐induced charge dissipation, ensuring robust performance under practical physiological and environmental conditions.

### 2.3 Shielding‐Enabled Interference‐Resilient Sensor‐Circuit Design

**FIGURE 3:**
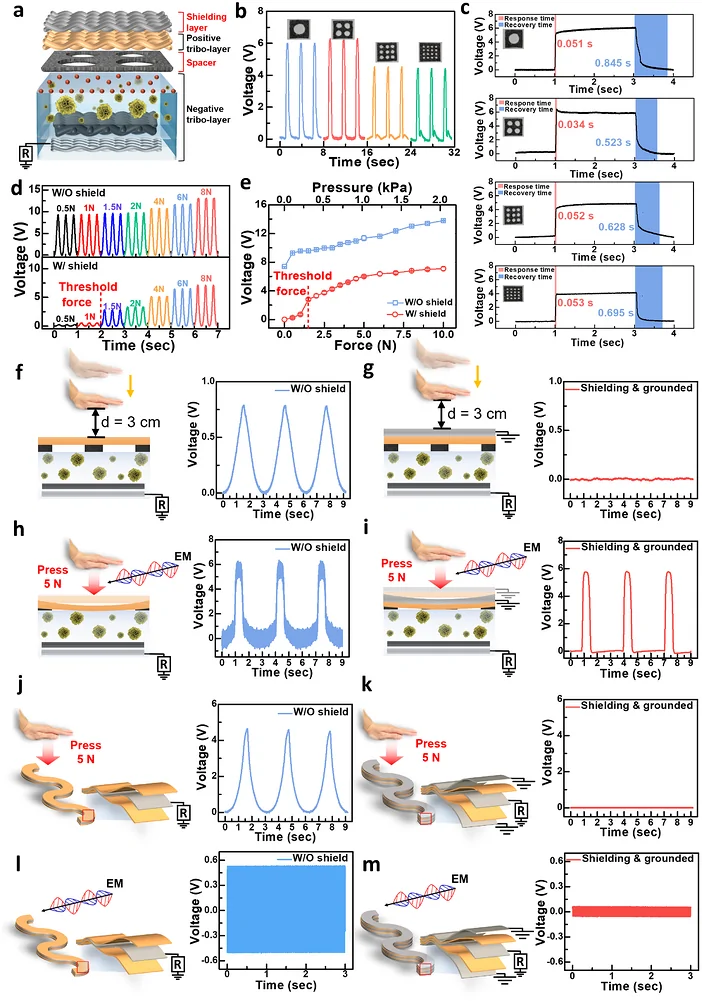
Electrical and EM shielding of sensing unit and circuit. (a) Schematic structure of flexible triboelectric sensor. (b) Differences in voltage signals generated by spacers with different hole arrays. (c) Effect of spacers with different hole arrays on response and recovery time. (d) Voltage signals and (e) voltage–pressure/force relationship of triboelectric sensors with and without shielding design. The dashed line indicates the detection threshold used to determine the minimum force for reliable signal recognition. Voltage signals generated by triboelectric sensors (f) without and (g) with shielding, under non‐contact back‐and‐forth hand movement. Voltage signals generated by triboelectric sensors (h) without shielding and (i) with shielding, under a pressing force of 5 N and electromagnetic interference. Voltage signals generated on signal transmission circuits (j) without and (k) with shielding, under a pressing force of 5 N. Voltage signals generated by signal transmission circuits (l) without shielding and (m) with shielding, under an EM interference.

The triboelectric sensing unit converts mechanical motion into electrical signals through periodic contact‐separation of the triboelectric layers. To ensure flexibility, light weight, and wearing comfort, a porous sponge spacer was employed as the intermediate layer (Figure 3a). Its elastic and compressible structure maintains a stable air gap, enabling independent movement of the positive and negative tribo‐layers and facilitating efficient charge separation during repeated deformation.

Spacer geometry was further optimized using circular hole arrays with identical effective contact areas. The number of holes critically affects signal generation because the mechanical stiffness and contact convergence of the triboelectric layers depend on the structural integrity of the spacer. Single‐hole and 2 × 2 designs retain higher stiffness and better recoverability, allowing better contact between layers and producing stronger, cleaner voltage outputs (Figure 3b). In contrast, 3 × 3 and 4 × 4 configurations are mechanically weaker and deform nonuniformly, reducing effective contact and increasing signal noise.

Subsequently, the dynamic characteristics of the sensors were evaluated to identify the optimal spacer configuration for motion‐resolved signal transduction (Figure 3c). Under a 5 N loading condition, the 2 × 2 hole array exhibited the fastest response and recovery behavior, with a response time of 0.034 s and a recovery time of 0.523 s, outperforming the other geometries. The improved dynamics reflect the balanced mechanical support and deformation kinetics of the 2 × 2 structure, which enables rapid contact–separation and efficient charge release. Long‐term stability tests further confirm the robustness of this design. The device maintained consistent voltage outputs over 1000 operation cycles and after six washing processes (Figure S19), demonstrating excellent operational durability for wearable applications. On the basis of its superior dynamic response and reliability, the 2 × 2 hole array spacer was selected as the optimal configuration for subsequent system integration.

In triboelectric systems, the output can be easily perturbed by nearby dielectrics, including clothing and the human body, leading to severe signal misrecognition [16]. A conductive shielding layer was introduced above the positive tribo‐layer to eliminate the sensing interface and improve system reliability [22]. As shown in Figure 3d,e, devices without shielding generate voltages of ≈10 V under a small force of 0.5 N due to contactless electrostatic induction, which superimposes on the genuine press‐induced output. In contrast, the shielded device exhibits a clear linear force‐signal relationship by eliminating these parasitic charges. Simulations corroborate that a charged object approaching within 10–30 mm induces a nearly identical surface field, whereas the shielding layer almost completely blocks this field (Figure S20).

Notably, the shielding layer increases the activation threshold from ≈0.5 to ≈1.5 N, which is advantageous in wearable scenarios. The threshold force was defined from the voltage–force relationship in Figure 3e as the minimum force required to generate a distinguishable triboelectric signal above the detection level under periodic contact–separation operation. As further confirmed in Figure S21, the threshold remains consistently above ≈1.5 N across different operation frequencies (1–3 Hz), indicating negligible dependence on contact speed. In addition, the threshold behavior is governed by the compressive deformation of the sponge spacer that enables effective contact–separation between triboelectric layers, and is therefore independent of device geometry within the same structural configuration. Body micro‐motions, garment friction, and airflow fluctuations typically generate sub‐newton disturbances that would trigger false responses in unshielded devices. By raising the threshold, the sensor responds only to intentional mechanical inputs, significantly enhancing signal selectivity and stability during practical use.

In single‐electrode TENGs, severe false outputs are often induced by contactless electrostatic coupling when nearby objects move, leading to significant signal misinterpretation [21]. To assess the effectiveness of the shielding layer, a hand was moved back and forth at a distance of 3 cm from the device without physical contact (Figure 3f,g; Figure S22). In the unshielded configuration, this non‐contact disturbance generated noise signals of 0.75 V. Adding a shielding layer reduced the noise to 0.22 V, corresponding to a misrecognition rate *R*_m_ = *V*_m_ /*V*_0_. *V*_0_ was the initial voltage value and *V*_m_ was the voltage value of misrecognition signals. Therefore, *R*_m_ describes the fraction of the original unshielded interference signal that remains after shielding. In the non‐contact hand‐motion test, *V*_0_ was approximately 0.75 V, while *V*_m_ decreased to 0.22 V after shielding and further to 0.001 V after grounding the shielding layer, corresponding to *R*_m_ values of approximately 0.29 and 0.13%, respectively. These results quantitatively demonstrate the shielding design reduces misrecognition by nearly three orders of magnitude, greatly enhancing the robustness of the sensing system in practical wearable scenarios.

In addition to electrostatic disturbances, surrounding electrical appliances generate EM waves that introduce noise into triboelectric outputs, which also severely interferes with signal identification and learning in large‐area, full‐body sensing [17]. To assess EM immunity, a laptop was placed beneath the device at a distance of ≈5 cm while applying a 5 N contact‐separation motion (Figure 3h,i; Figure S23). In the unshielded configuration, EM interference produced a noise amplitude of 0.8 V, whereas the shielded device reduced this noise to 0.001 V, corresponding to an attenuation factor of ≈800 and a shielding effectiveness of ≈58 dB. This substantial suppression demonstrates that the shielding layer provides strong EM isolation and preserves signal fidelity in wearable environments rich in electronic devices.

The introduction of the shielding layer significantly improves the clarity of the contact‐separation signals, enabling more reliable downstream interpretation. In addition, the recovery time is markedly reduced from 0.523 to 0.108 s (Figure S24). This acceleration arises from the suppression of parasitic electrostatic induction: without shielding, residual charges induced by external objects prolong the relaxation process after contact, whereas the shielding layer minimizes these residual fields, allowing the device to return to its baseline state more rapidly.

Despite the advantages of TENG‐based sensing arrays, a critical challenge arises from contact‐induced misrecognition on the circuit, in which signals generated on densely routed conductive wires are mistakenly interpreted as legitimate sensing outputs [22]. This problem is intrinsic to large‐area and multiplexing triboelectric systems: body movement, garment deformation, or incidental contact can induce charges directly on the wiring rather than on the sensing nodes, making it impossible to distinguish circuit artifacts from true tactile signals.

To address this, shielding of the signal‐transmission interconnects becomes essential. When a 5 N force was applied to the unshielded circuit, a peak voltage of 4.7 V was observed (Figure 3j), demonstrating that even modest mechanical disturbances on the wiring can generate pronounced parasitic outputs. These circuit‐induced signals fundamentally limit the scalability and reliability of multiplexed wearable TENG systems. We therefore engineered a multilayer shielded‐interconnect architecture composed of conductive fabric (shielding layer), polyester fiber (insulation), conductive fabric (electrode), polyester fiber (insulation) and an outer conductive‐fabric shielding layer. This laminate structure eliminates charge coupling from external motion, suppresses parasitic electrostatic induction on the wiring, and minimizes EM interference (Figure 3k–m; Figures S25 and S26).

By electrically isolating the functional electrode from environmental and body‐induced disturbances, the multilayer shielding design ensures that only signals originating from the intended sensing nodes propagate through the circuit. This system‐level strategy is crucial for achieving artifact‐free signal acquisition in large‐area wearable arrays and represents a foundational step toward scalable, full‐body, and high‐fidelity triboelectric sensing.

### 2.4 DL‐Assisted Interpretation of Full‐Body Signals for Interactive Control

#### 2.4.1 Upper‐Body Gesture Decoding for Presentation Interaction

The large‐area multiplexed sensing garment, enabled by the shielded sensor‐circuit architecture, forms a high‐fidelity whole‐body motion acquisition platform that can capture subtle joint dynamics with minimal interference. Unlike conventional TENG‐based wearables, often limited by circuit‐induced artifacts and sparse sensing layouts, our system ensures that signals originate exclusively from designated sensor nodes, allowing full‐body gestures to be mapped with high reliability across eight distributed channels.

**FIGURE 4:**
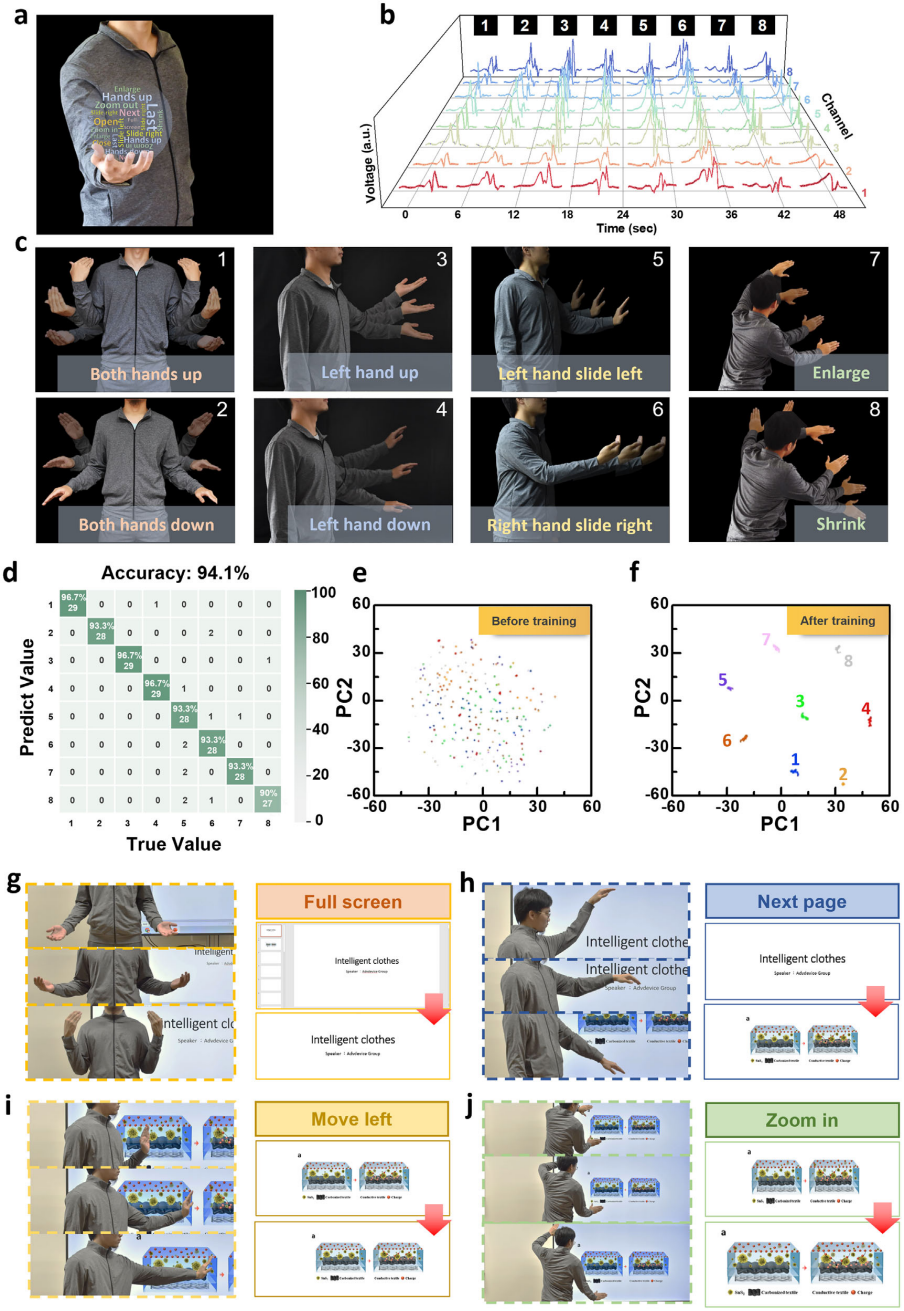
DL‐assisted upper‐body motion recognition for presentation interaction. (a) Schematic illustration of operating through smart garments. (b) Layout of the eight triboelectric sensing units. (c) Representative multichannel voltage signals generated by human motion, accompanied by corresponding optical images of the gesture set. (d) Confusion matrix summarizing the classification accuracy of the trained DL model. (e) PCA of raw gesture data prior to training, exhibiting substantial class overlap. (f) PCA of learned feature representations after training, showing well‐separated gesture clusters. (g–j) Demonstrations of body movements mapped to specific presentation‐control commands, including full‐screen activation, slide navigation, content translation, and zoom control.

As shown in Figure 4a–c, the garment‐scale sensing platform captures upper‐body gestures through eight distributed triboelectric nodes that collectively record distinct spatiotemporal voltage patterns. The predefined gesture set includes both hands up, both hands down, left hand up, left hand down, left hand slide left, right hand slide right, enlarge, and shrink, each producing a characteristic multi‐channel response that enables reliable differentiation across the eight classes. After model training, the system achieves a classification accuracy of 94.6% (Figure 4d), with clear inter‐class separation revealed by Principal Component Analysis (PCA) and t‐distributed stochastic neighbor embedding (t‐SNE) analyses (Figure 4e,f). Dimensionality‐reduction analyses further reveal the high separability of the learned feature space: while raw data show substantial inter‐class overlap (Figure 4e), the trained representations form compact, clearly segregated clusters (Figure 4f), underscoring the discriminative power enabled by the multi‐node, full‐body sensing configuration.

This gesture‐decoding framework transitions seamlessly into practical human–computer interaction. As demonstrated in Figure 4g–j and Movie S1, raising both hands activates full‐screen mode, an upward sweep advances slides, lateral arm motion controls content translation, and bi‐manual expansion or contraction gestures adjust zoom levels. Such versatility arises from the synergistic integration of a high‐density whole‐body sensing network, the shielding‐reinforced circuit architecture that ensures artifact‐free signal acquisition, and a modular deep‐learning pipeline operating on the on‐garment processor. Collectively, these features establish a scalable and robust platform capable of supporting presentation control, rehabilitation training, and immersive metaverse applications.

To further demonstrate upper‐body motion decoding, five characteristic throwing angles (60°, 30°, 0°, ‐30°, ‐60°) were selected as test gestures (Figure S27 and Movie S2). The spatially distributed sensors capture coordinated shoulder–elbow–wrist dynamics, generating multidimensional temporal signatures that form robust feature spaces for the DL model. This sensing architecture enables real‐time interaction with a throwing‐simulation game. Joint‐level motion patterns are translated into continuous control of the duck's flight trajectory, demonstrating seamless end‐to‐end integration from on‐body biomechanical sensing to wireless data transmission, DL inference, and interactive metaverse‐style actuation.

#### 2.4.2 Lower‐Body Motion Decoding for Interactive Soccer Control

Building on the validated capability for upper‐body gesture decoding and interactive control, we then evaluated the generality of the sensing garment by extending motion recognition to lower‐limb activities. This progression from upper‐limb gestures to lower‐limb motions shows the system's capacity to adapt its sensing to different body regions. Lower‐body motions produce complex multi‐joint dynamics and larger variations in mechanical loading, offering a stringent test for the robustness and scalability of the sensing‐circuit‐algorithm pipeline.

**FIGURE 5:**
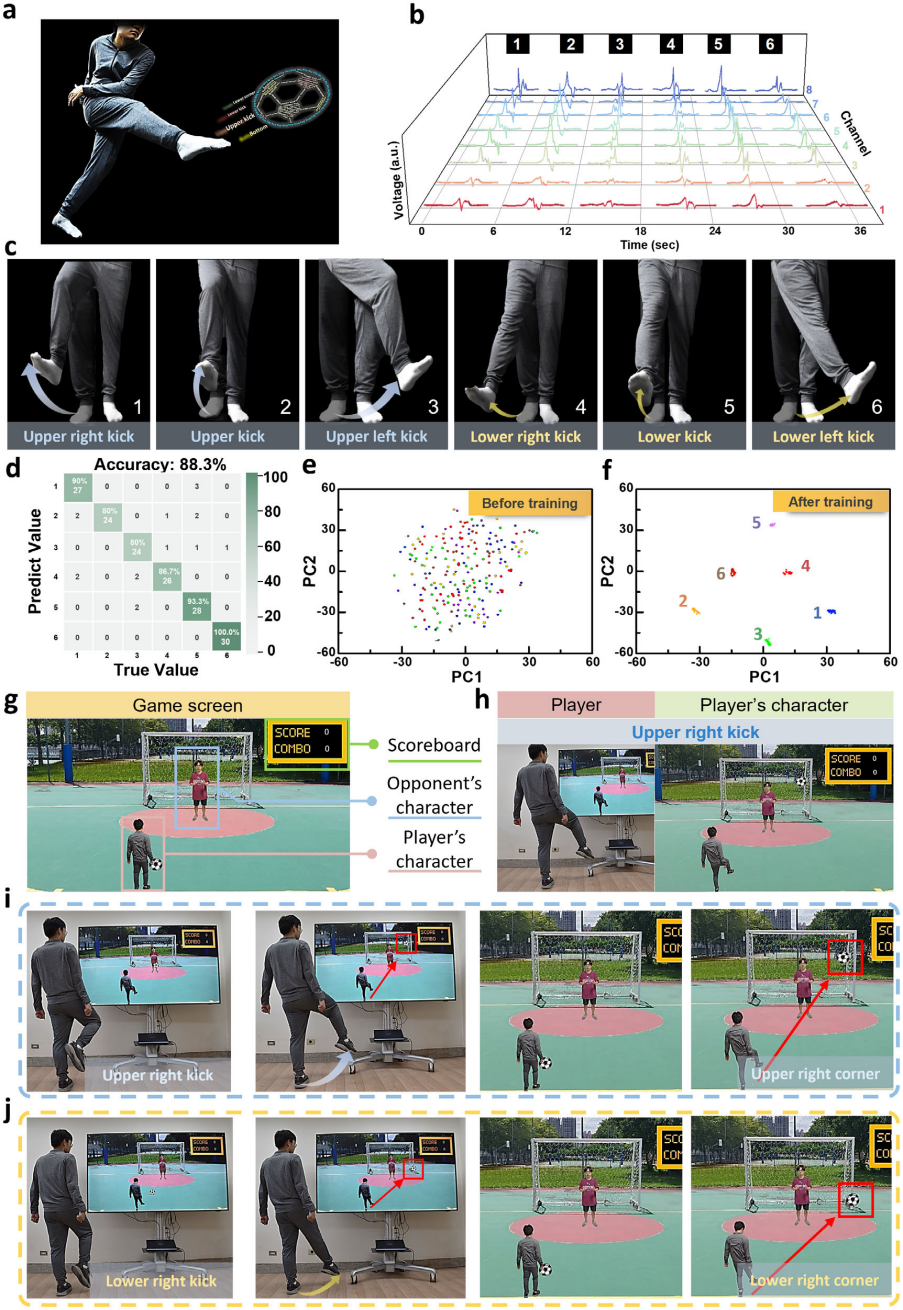
Smart garment‐enabled lower‐body motion decoding for interactive soccer control. (a) Schematic illustration of playing throwing game in sensing clothes. (b) Sensor number and (c) data generated by lower‐body motion and optical graphs of lower‐body motion corresponding to the number. (d) Accuracy of confusion matrix for learned model identification of lower‐body motions in playing soccer. Visualized principal component analysis (e) before and (f) after training. Manipulation of (g) game screen and (h) gameplay elements for player and player's character. (i) and (j) Demonstration of lower‐body movements mapped to specific presentation control commands in playing soccer game.

To evaluate lower‐limb sensing and classification, the sensing garment was used to capture electrical signatures generated during a series of kicking gestures (Figure 5a). Differences in kick height, direction, and angular trajectory yield distinct signal patterns across the multi‐node network, enabling accurate decoding of leg movements. Representative signals from six kick types are shown in Figure 5b,c, accompanied by the corresponding optical motion frames. After training, the DL model achieved an accuracy of 88.3%, with well‐separated clusters in PCA and t‐SNE space (Figure 5d–f), demonstrating robust feature extraction despite the higher mechanical intensity of lower‐body motions.

These trained motion classes were integrated into a virtual soccer game, enabling real‐time control of shooting direction (Figure 5g–j, Figure S28 and Movie S3). Upper‐right kicks consistently guided the ball toward the upper‐right corner of the goal (Figure 5i), whereas lower‐right kicks directed shots toward the lower‐right corner (Figure 5j). This direct mapping between complex leg gestures and precise in‐game actions reveals the system's potential for interactive sports training, rehabilitation, and immersive metaverse environments.

#### 2.4.3 Full‐Body Action Decoding for Immersive Fighting‐Game Interaction

To further validate the upper limits of our system, we selected one of the most challenging scenarios in human–computer interaction: real‐time recognition of full‐body, multi‐joint, high‐speed fighting actions. Unlike the relatively structured upper‐body angles (Figure 4) or the directional lower‐limb kicks (Figure 5), these gestures involve rapid and large‐area biomechanical transitions, wide angular excursions, asymmetric limb coordination, and high inter‐trial variability. These movements occur within tens of milliseconds, engage the entire body, and involve complex combinations of translational and rotational components. Under such conditions, most wearable systems fail because of signal mixing, circuit interference, and insufficient temporal resolution. Reliable decoding therefore demands high‐density full‐body sensing, interference‐free signal pathways, stable wireless transmission, and a DL model that remains robust to nonlinear, high‐dimensional features.

**FIGURE 6:**
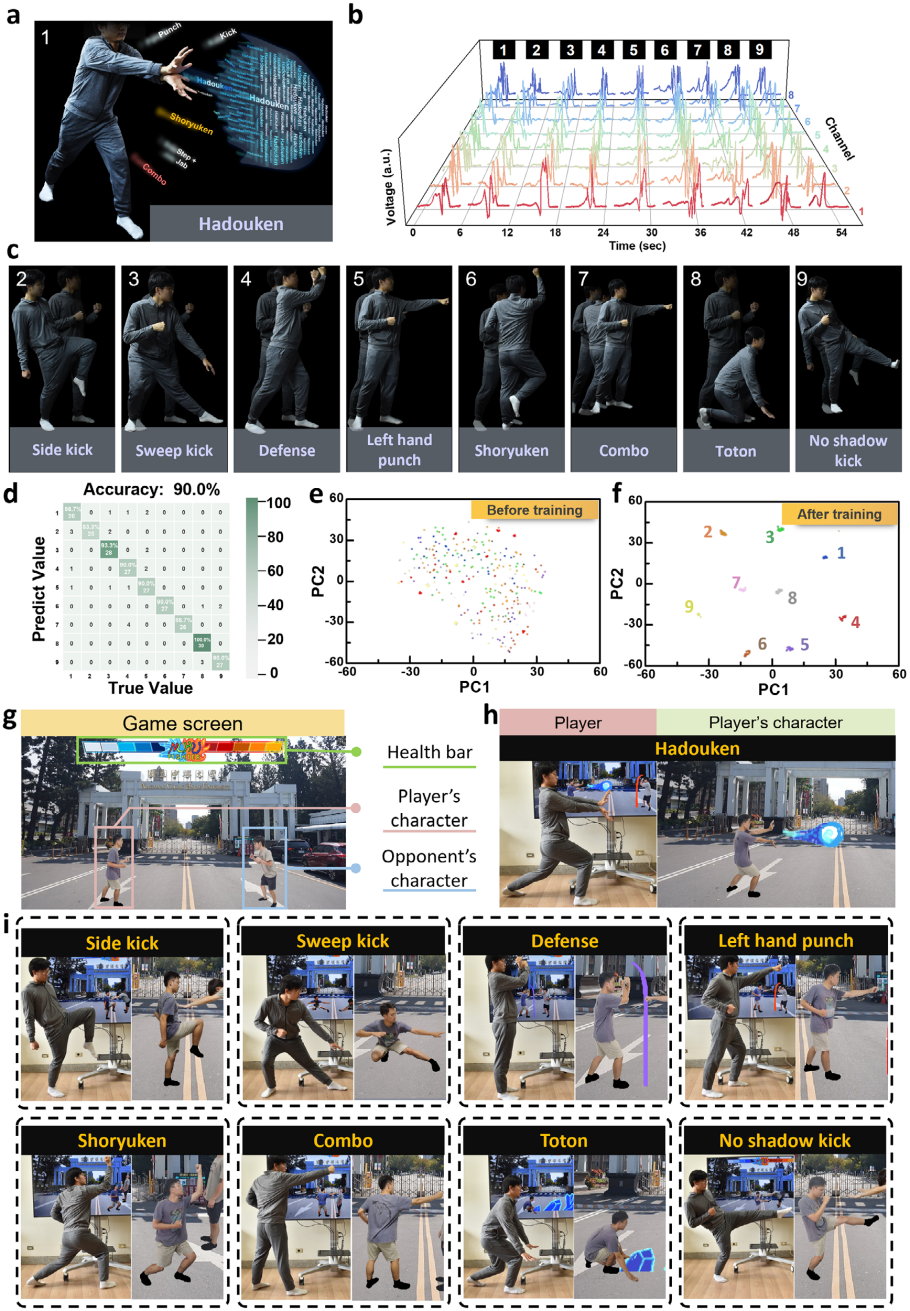
Full‐body motion recognition for virtual fighting control. (a) Schematic illustration of the sensing garment controlling a fighting game. (b) Sensor indexing and (c) representative voltage signals collected from full‐body motions, along with corresponding optical images. (d) Confusion matrix showing classification accuracy of nine distinct fighting actions. (e, f) PCA visualization of motion features before and after model training. (g) Control of game interface and (h) execution of in‐game actions using decoded body gestures. (i) Demonstrations of full‐body movements mapped to specific combat commands.

To push the sensing platform to its highest level of complexity, we applied the sensing garment to control a “*Street Fighter*”—style virtual combat system, requiring recognition of nine full‐body gestures that encompass offensive, defensive, and combination movements (Figure 6a–c). These actions involve rapid transitions, spatially asymmetric limb trajectories, and coordinated rotations of the torso and extremities, conditions under which most wearable sensing systems lose fidelity. Despite these challenges, the system achieved a 90.0% classification accuracy across the nine action classes (Figure 6d), with PCA and t‐SNE visualizations showing clear post‐training separation (Figure 6e,f). This validates the robustness of the shielding‐enhanced large‐area sensing network architecture during intense and full‐body movement.

A rule‐based decision engine was further incorporated to translate physical gestures into in‐game tactical logic. For instance, “*Toton*” successfully defends against “*No shadow kick*,” while “*No shadow kick*” counters “*Shoryuken*” creating a bidirectional interaction strategy (Figure S29 and Movie S4). By performing the corresponding gestures, players can control the in‐game characters to execute corresponding moves (Figure 6g–i; Figure S30).

As shown in Figure S31, the shielding strategy significantly improves system‐level motion recognition performance across upper‐body, lower‐body, and full‐body scenarios by effectively suppressing parasitic electrostatic induction and electromagnetic interference in large‐area multiplexed triboelectric sensing systems. This demonstration highlights the expressive capability and scalability of the smart sensing garment by showing its ability to decode rapid full‐body actions and translate them into complex virtual interactions. The system thus provides an effective and immersive interface for gaming, sports training, rehabilitation, and metaverse‐oriented human–machine interaction.

To further evaluate subject‐to‐subject robustness, multi‐subject validation was performed using three testers with different body sizes: Tester 1, 179 cm/80 kg; Tester 2, 165 cm/70 kg; and Tester 3, 185 cm/94 kg. As shown in Figure S32, the sensing garment maintains reliable recognition performance across upper‐body, lower‐body, and full‐body motion scenarios for all testers.

In addition to sensing accuracy and interactive capability, practical wearability is essential for garment‐scale motion‐capture systems. The sensing garment is constructed from textile‐compatible components, including conductive fabrics, polyester fabrics, sponge spacers, and serpentine textile interconnects, which help preserve softness, flexibility, and breathability during body motion. The distributed sensing nodes and waist‐mounted wireless module reduce interference with natural movement, while the serpentine circuits accommodate repeated bending and stretching. Together with the stable output after 1000 operation cycles and six washing processes, these results support the garment‐level durability and practical wearability of the system. Future work will further optimize breathable encapsulation, electronic miniaturization, and long‐term user‐wear comfort under daily‐life conditions.

## 3 Conclusions

This work presents a garment‐scale triboelectric sensing platform that enables reliable full‐body motion capture through large‐area, multi‐channel sensing networks integrated within noise‐free stretchable circuits. A unified multilayer shielding architecture that integrates circuit‐level interference shielding, EM suppression, and controlled threshold voltage conditions ensures low‐latency and high‐fidelity whole‐body signal capture during complex dynamic motions. When coupled with on‐body wireless transmission and a DL algorithm, the system achieves accurate decoding of multi‐site biomechanical signals and supports various immersive applications. By enabling interference‐resilient sensing at garment scale, this shielding strategy establishes a generalizable materials‐to‐system framework for full‐body motion capture, supporting continuous tracking of multi‐joint coordination and whole‐body dynamics beyond the capability of localized sensors. Such functionality is critical for precise characterization of natural movement, high‐resolution behavioral analysis, and more intuitive human–machine interactions. Future advances in multimodal sensing and adaptive learning algorithms will further enhance full‐body motion capture and expand its impact in smart sensing garments, the metaverse, and related applications.

## 4 Experimental Section

### 4.1 Preparation of Graphite‐Like Carbonized Textile

Pure cotton fabric without dye or fluorescent agents was selected as the raw material. The fabric was rinsed with deionized water to remove surface and internal impurities and then dried. After drying, it was cut into appropriate sizes and placed into a tube furnace. The furnace was first evacuated using a vacuum pump, then purged with nitrogen gas until fully inert. Nitrogen was continuously introduced at a flow rate of 3 sccm. Thermal treatment was conducted in three stages: (1) Heating to 900°C at a rate of 3°C min^−1^; (2) Maintaining at 900°C for 200 min; (3) Cooling to room temperature (30°C) within 20 min. The resulting material was the graphite‐like carbonized textile, used as the charge storage layer.

### 4.2 Synthesis of SnS_2_ NFs

SnS_2_ NFs were synthesized via a hydrothermal method. Specifically, 1.77 g of tin (IV) chloride (SnCl_4_) and 1.15 g of thioacetamide (TAA) were dissolved in a solvent mixture of 65 mL isopropanol (IPA) and 5 mL deionized water, and stirred magnetically for 15 min. The solution was transferred to a Teflon‐lined autoclave and heated at 200°C for 22 h. After naturally cooling to room temperature, the product was washed several times with DI water and ethanol using centrifugation and ultrasonic agitation. The final product was dried in an oven at 60°C for several hours to obtain SnS_2_ nanoflower powder.

### 4.3 Fabrication of the Sensing Device

SnS_2_ NFs powder was mixed with silicone rubber at a concentration of 0.2 wt%. The mixture was poured into a 7 × 7 cm^2^ mold featuring a sandpaper‐templated surface. A layer of carbonized textile was placed onto the mold to serve as the charge storage layer, followed by a conductive fabric layer as the electrode. After the silicone rubber cured, a sponge and polyester fiber were sequentially added as the isolation and triboelectric layers, respectively. The entire device was then encapsulated with conductive fabric to shield external noise.

### 4.4 Fabrication of Stretchable Electrodes

The stretchable electrode consists of a five‐layer structure arranged as follows (top to bottom): conductive fabric, polyester fiber, conductive fabric, polyester fiber, and conductive fabric. The top and bottom conductive fabric layers serve as shielding layers, the polyester fiber layers as insulators, and the central conductive fabric as the working electrode. A laser cutter was used to pattern the structure into a serpentine shape.

### 4.5 Fabrication of the Motion‐Sensing Garment

Sensing units were sewn onto key joint areas of the body—shoulders, elbows, wrists, and knees. During body movements, mechanical deformation of the joints induced signals from the corresponding sensors. These signals were transmitted via onboard circuitry to a microcontroller unit and then sent via Bluetooth to a computer for machine learning‐based signal interpretation.

### 4.6 Hardware Specification and System Configuration

The on‐body motion‐sensing system is implemented using an Arduino Mega 2560 (ATmega2560, 8‐bit AVR microcontroller, 16 MHz) as the central processing unit for triboelectric signal acquisition and control. The system operates at a sampling rate of 1000 data points per second and supports wireless data transmission via a 9600 bps Bluetooth module to a host computer for further analysis. The average power consumption of the microcontroller is approximately 5 V and 50–70 mA, and the entire system is powered by a 10 000 mAh portable power bank, enabling long‐term continuous operation. The system exhibits an overall latency of approximately 2 s, which originates from the post‐processing‐based deep‐learning strategy in which complete motion cycles are collected prior to classification to ensure robust recognition performance.

### 4.7 Data Collection and Dataset Configuration

After the electrical signals were transmitted to the computer, a convolutional neural network‐based DL model was employed to analyze the data. Specifically, 100 sets of motion waveforms are converted into images and subsequently processed through convolution, pooling, flattening, and classification layers to generate a classifier capable of interpreting body movements. This enabled real‐time interaction and control through physical motion in real‐world applications. To enhance the accuracy of the model, several parameters were optimized, including the number of sensor channels, kernel size, stride, number of filters, and number of convolutional layers. The highest classification accuracy was achieved when using 8 sensor channels, a kernel size of 7, a stride of 3, excluding the use of 256 filters, and avoiding a four‐layer convolutional architecture (Figure S33).

### 4.8 Characterization and Measurement

SEM (ZEISS ULTRA PLUS), energy‐dispersive X‐ray spectroscopy (EDS, OXFORD X‐Max 50 mm^2^), XRD (Rigaku Ultima IV), Raman scattering spectroscopy (UniDRON), and field‐emission TEM (TEM, Tecnai G2) were employed to explore the morphologies, valence states, crystal structures, components, and crystallinities of the as‐synthesized GT, SnS_2_ NFs, SnS_2_ NFs‐SR composites. *V*_oc_, *Q*_tr_, and charging curves were measured using a Keithley 6517A electrometer/high‐resistance meter. *I*_sc_ was measured using a Stanford low‐noise current amplifier (model SR570). A National Instruments BNC‐2120 device was used as a junction box for the physical connection of the external signal to be tested, and a National Instruments PCI‐6259 multifunction device was used for data collection. A Trek Model 542A electrostatic voltmeter was used to measure the static voltage data for the determination of the maximum surface potential and potential degradation over time. LabVIEW was used as the software platform for data acquisition, analysis, prediction, and feedback commands.

## Author Contributions

**Tzu‐Ching Lu**: methodology and software. **Wei‐Chun Yang**: methodology, formal analysis, and data curation. **Ruiyuan Liu**: writing – review and editing and supervision. **Wei‐Chen Peng**: methodology, software, and data curation. **Jiann‐Yeu Chen**: data curation and software. **Zhi‐Xian Yan**: validation and investigation. **Tai‐Chen Wu**: investigation and validation. **Jiun‐Wei Fong**: methodology and software. **Beibei Shao**: conceptualization, writing – original draft, writing – review and editing, and investigation. **Kai‐Yuan Hsiao**: investigation and software. **Yi‐Ting Chen**: methodology, investigation, data curation, and formal analysis. **Heng‐Jui Liu**: supervision and writing – review and editing. **Ying‐Chih Lai**: supervision, conceptualization, and writing – review and editing. **Ming‐Yen Lu**: software and data curation. **Ming‐Han Lu**: methodology, software, and data curation.

## Funding

This work was supported by the National Science and Technology Council, Taiwan grant 114‐2628‐E‐005‐002; 114‐2112‐M‐005‐002; “Innovation and Development Center of Sustainable Agriculture” from The Featured Areas Research Center Program within the framework of the Higher Education Sprout Project by the Ministry of Education (MOE) in Taiwan;National Natural Science Foundation of China grant (52103306, 22408248); The Collaborative Innovation Center of Suzhou Nano Science and Technology; 2025 Independent Research Project of the Jiangsu Provincial Key Laboratory of Carbon‐Based Functional Materials and Devices for High‐Tech Research (ZZ2506).

## Conflicts of Interest

The authors declare no conflicts of interest.

## Supporting information




**Supporting File 1**: advs77038‐sup‐0001‐SuppMat.docx.

**Supporting File 2**: advs77038‐sup‐0002‐MovieS1‐S4.zip.

## Data Availability

The data that support the findings of this study are available from the corresponding author upon reasonable request.
