# Supplementary material for: Large‐Area Noise‐Resilient Multiplexing Triboelectric Biomechanical Sensing on Clothing for High‐Precision Full‐Body Motion Capture in Wearables
Source: Adv Sci (Weinh). 2026 Aug 6;13(60):e77038. doi: 10.1002/advs.77038 (PMC13445311; doi:10.1002/advs.77038)
Supplement: Supplementary file 1 [file ADVS-13-e77038-s001.docx]

Supplementary Information

**Large-area noise-resilient multiplexing triboelectric biomechanical sensing on clothing for high-precision full-body motion capture in wearables**

**This PDF file includes:**

Figs. S1 to S33

Supplementary Figures 1 to 33

Table S1

Legends for Movies S1 to S4

**Other Supplementary Materials for this manuscript include the following:**

Movies S1 to S4

**Supporting Information 1. Comparison with state-of-the-art TENG-based wearable motion-sensing systems.**

**Table S1.** Comparison of this work with representative state-of-the-art TENG-based wearable motion-sensing systems.

| TENG materials | Sensing nodes | Flexibility | Wireless capability | Interference resilience | Accuracy | Full body control | Ref. |
| --- | --- | --- | --- | --- | --- | --- | --- |
| SR/SnS_2_/SnS_2_-doped GT | 8 | ○ | ○ | ○ | 94.6% | ○ | This work |
| Ecoflex/wrinkled nitrile | 15 | ○ | ╳ | ○ | 91.3% | ╳ | [1] |
| silicone rubber | 5 | ○ | ○ | ○ | 94% | ╳ | [2] |
| V₂CT_x_/silicone nanocomposite | 5 | ○ | ○ | N/A | N/A | ╳ | [3] |
| roughened chitosan/FEP | 14 | ○ | ╳ | ╳ | N/A | ╳ | [4] |
| PTFE rollers/copper | 2 | ╳ | ○ | N/A | 99.91% | ╳ | [5] |
| carbon-doped silicone rubber | 4 | ○ | ╳ | ○ | 92.63% | ╳ | [6] |

**Supporting Information 2. Working mechanism of the triboelectric sensor**

**Fig. S1.** Sensing and shielding process of the triboelectric sensor.

**Supporting Information 3. Flowchart for operating the smart garment**

**Fig. S2.** Pipeline for motion sensing, wireless data transmission, deep-learning inference, and real-time action recognition.

**Supporting Information 4. Material characteristics of SnS_2_ NFs**

**Fig. S3.** EDS mapping of (**a**) Sn element and (**b**) S element, and (**c**) XRD patterns of the synthesized SnS_2_ NFs.

**Supporting Information 5. Material characteristics of SnS_2_ NFs**

**Fig. S4.** **TEM and HRTEM characterization of the synthesized SnS_2_ NFs.** (**Top**) Low-magnification TEM images show uniform SnS_2_ NFs with well-defined lateral morphology. (**Bottom**) High-resolution TEM reveals clear lattice fringes, with measured spacings of 0.28 nm and 0.19 nm corresponding to the (101) and (120) crystal planes of SnS_2_ NFs, confirming their high crystallinity.

**Supporting Information 6. Material characteristics of SnS_2_ NFs**

**Fig. S5.** (**a)** XPS survey spectrum of the SnS_2_ NFs. (**b**) High-resolution S 2p spectrum with peak deconvolution. (**c**) High-resolution Sn 3d spectrum with fitted components.

**Supporting Information 7. Material characteristics of the SR**

**Fig. S6.** SEM image of the prepared SR molded on sandpaper.

**Supporting Information 8. Material characteristics of SnS_2_ NFs-SR composite**

**Fig. S7.** SEM image of the SnS_2_ NFs doped into SR.

**Supporting Information 9. Material characteristics of the GT**

**Fig. S8.** (**a**) SEM image of pristine textile. Corresponding SEM elemental mapping images of (**b**) C and (**c**) O elements. (**d**) SEM image of the GT. Corresponding SEM elemental mapping images of (**e**) C and (**f**) O elements. (**g**) Comparison of the weight percentages of the pristine textile and the GT.

**Supporting Information 10. Material characteristics of the GT**

**Fig. S9.** Raman spectra of the pristine textile and the GT.

**Supporting Information 11. Material characteristics of the GT**

**Fig. S10.** (**a)** XPS survey spectrum of the GT. (**b**) High-resolution C 1s spectrum with peak deconvolution. (**c**) High-resolution O 1s spectrum with fitted components.

**Supporting Information 12. Material characteristics of the GT**

**Fig. S11.** **TEM and HRTEM characterization of the GT.** (**Left**) Low-magnification TEM image shows the morphology of the GT nanosheet, with the inset displaying its corresponding SAED pattern, indicating high crystallinity. (**Right**) High-resolution TEM reveals clear lattice fringes with an interlayer spacing of 0.35 nm.

**Supporting Information 13. Material characteristics of Ag-fiber textile**

**Fig. S12.** (**a**) SEM and (**b**) EDS mappings of Ag-fiber textile.

**Supporting Information 14. Electrical output of the triboelectric sensor**

**Fig. S13.** Output (**a**) *I*_SC_ and (**b**) *V*_OC_ of the devices with different concentrations of SnS_2_ NFs.

**Supporting Information 15. Dielectric constant of the SnS_2_ NFs-doped SR**

**Fig. S14.** Effect of the concentrations of SnS_2_ NFs on the dielectric constant of the SnS_2_ NFs-doped SR.

**Supporting Information 16. Electrical output of the triboelectric sensor**

**Fig. S15.** Output (**a**) *V*_OC_ and (**b**) *I*_SC_ of the devices with different contact-electrification materials with a SnS_2_ NFs weight ratio of 0.2 wt%.

**Supporting Information 17. Electrical output of the triboelectric sensor**

**Fig. S16.** Output (**a**) *I*_SC_ and (**b**) *V*_OC_ of the devices with different configurations.

**Supporting Information 18. Schematic illustration of the contact-electrification mechanism**

**Fig. S17**. Scheme of energy bands of the polyester/SR/SnS_2_/SR/GT/SR device, illustrating interfacial electron transfer from the polyester to the SnS_2_ NFs-decorated GT.

**Supporting Information 19. Electrical outputs of the triboelectric sensor**

**Fig. S18.** (**a**) Output *I*_SC_ of the devices under different relative humidity. (**b**) Extracted peak *I*_SC_ showing a linear decay with increased humidity. (**c**) Extracted peak *V*_OC_ exhibiting a similar linear attenuation trend.

**Supporting Information 20. Electrical outputs of the triboelectric sensor**

**Fig. S19.** (**a**) Voltage output during 1,000 operation cycles. (**b**) Voltage retention after repeated operation cycles. (**c**) Voltage retention after six washing processes. Error bars represent the standard deviation from repeated measurements.

**Supporting Information 21. COMSOL simulations of the triboelectric sensor**

**Fig. S20.** Potential simulations of the devices (**a, b**) without and (**c, d**) with shielding design.

**Supporting Information 22. Electrical outputs of the triboelectric sensor**

**Figure S21**. Threshold force measurement under at (**a**)1 and (**b**) 2 Hz, showing frequency-independent behavior.

**Supporting Information 23. Electrical outputs of the triboelectric sensor**

**Fig. S22.** Voltage signal in non-contact mode (at a distance of 3 cm) of the device with shielding and no grounded design.

**Supporting Information 24. Electrical outputs of the triboelectric sensor**

**Fig. S23.** Voltage signals under pressure (5 N) and electromagnetic interference of the device with shielding and ungrounded design.

**Supporting Information 25. Electrical outputs of the triboelectric sensor**

**Fig. S24.** The response and recovery time of the device with a ground shielding layer.

**Supporting Information 26. Electrical outputs of the triboelectric sensor**

**Fig. S25.** Voltage signal of the circuit under pressure (5 N) with shielding and ungrounded.

**Supporting Information 27. Electrical outputs of the triboelectric sensor**

**Fig. S26.** Voltage signal of the circuit subjected to EM interference with shielding and ungrounded.

**Supporting Information 28. Motion-controlled slide-turning demo**

**Fig. S27. DL-based** **upper-body motion recognition for interactive game control**. **(a)** Schematic illustration of the throwing game operated using the sensing garment. The game is inspired by a campus scenario in which aggressive ducks roam the school grounds and attempt to chase or peck at passersby, requiring players to “throw” virtual objects to redirect or repel them. **(b)** Indexing of distributed sensors and **(c)** examples of motion-induced signals with corresponding optical images of associated gestures. **(d)** Confusion matrix showing classification accuracy of trained model for upper-body motion identification during the throwing game. **(e, f)** Visualization of feature space before and after model training using principal component analysis. **(g)** Real-time manipulation of game interface and **(h)** control of in-game character through decoded arm motions. **(i, j)** Demonstrations of the system accurately mapping 30° and –30° physical arm-throwing angles to the corresponding duck-throwing trajectories in the game, enabling precise interaction with the on-campus “attack duck” simulation.

**Supporting Information 29. Motion-controlled soccer-game demo**

**Fig. S28.** Extra demonstration of body movements mapped to specific control commands in playing a soccer game.

**Supporting Information 30. Motion-controlled fighting-game demo**

**Fig. S29.** Pictures showing the rules in playing a fighting game.

**Supporting Information 31. Motion-controlled fighting-game demo**

**Fig. S30.** Extra demonstration of body movements mapped to specific presentation control commands in playing a fighting game.

**Supporting Information 32. Training accuracies of the 2D CNN model**

**Figure S31.** (**a**) Upper-body, (**b**) lower-body, and (**c**) full-body motion recognition performance with and without shielding.

**Supporting Information 33. Training accuracies of the 2D CNN model**

**Figure S32.** **Multi-subject validation of DL-based motion recognition.** (**a**) Upper-body, (**b**) lower-body, and (**c**) full-body motion recognition performance of three testers.

**Supporting Information 34. Training accuracy–epoch curves of the 2D CNN model**

**Fig. S33. Training accuracy–epoch curves of the 2D CNN model.** (**a**) Convolutional neural network architecture diagram. Optimization of the model, including (**b**) the number of channels, (**c**) kernel size, (**d**) number of filters, and (**e**) number of convolutional layers.

**Supporting Information 35. Supplementary Movies**

**Supplementary Movie Legends**

**Movie S1**: The deep-learning-assisted smart garment enables presentation control by mapping compound upper-body gestures.

**Movie S2**: The deep-learning-assisted smart garment enables real-time interaction with a throwing-simulation game.

**Movie S3**: The deep-learning-assisted smart garment enables virtual soccer gameplay by mapping lower-body movements to real-time control of shooting direction.

**Movie S4**: The deep-learning-assisted smart garment controls a *Street Fighter*–style virtual combat system by recognizing nine full-body gestures.

**Supporting References**

[1] Wen, F., Zhang, Z., He, T., Lee, C. AI enabled sign language recognition and VR space bidirectional communication using triboelectric smart glove. *Nat. Commun.*, 2021, *12*(1), 5378.

[2] Sun, Z., Zhu, M., Shan, X., Lee, C. Augmented tactile-perception and haptic-feedback rings as human-machine interfaces aiming for immersive interactions. *Nat. Commun.*, 2022, *13*(1), 5224.

[3] Salauddin, M., Rana, S. S., Sharifuzzaman, M., et. al. Highly electronegative V_2_CT_x_/silicone nanocomposite‐based serpentine triboelectric nanogenerator for wearable self‐powered sensors and sign language interpretation. *Adv. Energy Mater.*, 2023, *13*(10), 2203812.

[4] Lu, D., Liu, T., Meng, X. et. al. Wearable triboelectric visual sensors for tactile perception. *Adv. Mater.*, 2023, *35*(7), 2209117.

[5] Hao, D., Fan, C., Xia, X. et. al. Hybrid electromagnetic‐triboelectric hip energy harvester for wearables and AI‐assisted motion monitoring. *Small*, 2025, *21*(21), 2500643.

[6] An, S., Pu, X., Zhou, S. et. al. Deep learning enabled neck motion detection using a triboelectric nanogenerator. *ACS nano*, 2022, *16*(6), 9359.
